# Cardioattentionnet: advancing ECG beat characterization with a high-accuracy and portable deep learning model

**DOI:** 10.3389/fcvm.2024.1473482

**Published:** 2025-01-06

**Authors:** Youfu He, Yu Zhou, Yu Qian, Jingjie Liu, Jinyan Zhang, Debin Liu, Qiang Wu

**Affiliations:** ^1^ Medical College, Guizhou University, Guiyang, Guizhou, China; ^2^ Department of Cardiology, Guizhou Provincial People’s Hospital, Guiyang, Guizhou, China; ^3^ Department of Cardiology, Guizhou Provincial Cardiovascular Disease Clinical Medicine Research Center, Guiyang, Guizhou, China; ^4^ Department of Cardiology, The Second Affiliated Hospital of Zunyi Medical University, Zunyi, Guizhou, China; ^5^ Department of Cardiology, First Affiliated Hospital of Dalian Medical University, Liaoyang, Liaoning, China; ^6^ School of Life Science and Technology, University of Electronic Science and Technology of China, Chengdu, Sichuan, China; ^7^ Department of Cardiology, The Second People’s Hospital of Shantou, Shantou, Guangdong, China

**Keywords:** cardiac arrhythmias, electrocardiogram, portable deep learning model, transformer model, Long Short-Term Memory, MobileNet

## Abstract

**Introduction:**

The risk of mortality associated with cardiac arrhythmias is considerable, and their diagnosis presents significant challenges, often resulting in misdiagnosis. This situation highlights the necessity for an automated, efficient, and real-time detection method aimed at enhancing diagnostic accuracy and improving patient outcomes.

**Methods:**

The present study is centered on the development of a portable deep learning model for the detection of arrhythmias via electrocardiogram (ECG) signals, referred to as CardioAttentionNet (CANet). CANet integrates Bi-directional Long Short-Term Memory (BiLSTM) networks, Multi-head Attention mechanisms, and Depthwise Separable Convolution, thereby facilitating its application in portable devices for early diagnosis. The architecture of CANet allows for effective processing of extended ECG patterns and detailed feature extraction without a substantial increase in model size.

**Results:**

Empirical results indicate that CANet outperformed traditional models in terms of predictive performance and stability, as confirmed by comprehensive cross-validation. The model demonstrated exceptional capabilities in detecting cardiac arrhythmias, surpassing existing models in both cross-validation and external testing scenarios. Specifically, CANet achieved high accuracy in classifying various arrhythmic events, with the following accuracies reported for different categories: Normal (97.37 ± 0.30%), Supraventricular (98.09 ± 0.25%), Ventricular (92.92 ± 0.09%), Atrial Fibrillation (99.07 ± 0.13%), and Unclassified arrhythmias (99.68 ± 0.06%). In external evaluations, CANet attained an average accuracy of 94.41%, with the area under the curve (AUC) for each category exceeding 99%, thereby demonstrating its substantial clinical applicability and significant advancements over traditional models.

**Discussion:**

The deep learning model proposed in this study has the potential to enhance the accuracy of early diagnosis for various types of arrhythmias. Looking ahead, this technology is anticipated to provide improved medical services for patients with heart disease through continuous, non-invasive monitoring and timely intervention.

## 1 Introduction

Cardiac arrhythmias, resulting from disruptions in the heart's electrical signals, frequently occur in clinical settings. Cardiac arrhythmias, which manifest in multiple forms with varying mechanisms and severities, are a major cause of morbidity and mortality (1). The China Hypertension Survey conducted from 2012 to 2015 reported a prevalence rate of 0.7% for atrial fibrillation in individuals aged 35 years and older, with 34.0% of these cases being undetected by the patients themselves (2). In the United States, most sudden cardiac deaths (SCDs) were due to ventricular tachyarrhythmias, making up 80% of these incidents (3). Bradyarrhythmias and conduction disorders can precipitate syncope, sudden cardiac death, and symptoms including fatigue and exercise intolerance due to intermittent heart rate inadequacy. However, these conditions can be difficult to identify (4).

Clinical screening for arrhythmia predominantly relies on patients presenting to healthcare facilities after the onset of symptoms. Under the supervision of specialized electrocardiographers or cardiologists, physicians are tasked with diagnosing arrhythmias. Furthermore, some patients may be identified as having various forms of arrhythmia during routine physical examinations (5). However, both detection methods necessitate a high degree of professionalism from healthcare providers. A cross-sectional study indicated that primary care physicians exhibited a misdiagnosis rate of 23% for abnormal electrocardiograms (6). It is noteworthy that, despite their expertise in cardiovascular medicine, the average diagnostic accuracy for ventricular tachycardia is only 78.4% (7). The large-scale training of specialized cardiologists is particularly impractical in rural regions worldwide, especially in underdeveloped countries. The advancement of artificial intelligence technology has facilitated the increased application of deep learning techniques for arrhythmia detection. Nonetheless, the integration of AI and deep learning into electrocardiography (ECG) detection poses distinct challenges due to the specialized nature of ECG interpretation.

Frameworks for interpreting electrocardiograms (ECGs), including parameters such as heart rate, rhythm, cardiac axis, intervals, and ventricular activity, have been developed to classify and identify various cardiac disorders in contemporary research. For instance, Attia et al. introduced a novel algorithm utilizing convolutional neural networks to predict paroxysmal atrial fibrillation (AF) in patients, achieving an overall accuracy of 83.3% based on benign, normal sinus rhythm ECGs (8). Similarly, Khurshid et al. (9). demonstrated that ECG *P*-waves significantly impacted the predictions made by artificial intelligence (AI) models during the training of convolutional neural networks. Their study employed a combined analysis of AI and clinical risk factor models for AF, which enhanced the functionality and accuracy of ECG-AI systems. Despite the relative robustness of these systems, the complex nature of the cardiac conduction system results in numerous ECG graphs exhibiting morphological characteristics that are not readily discernible to the human eye, complicating the identification of local morphology and inter-beat relationships. Furthermore, there remains a lack of comprehensive evaluation regarding the efficacy of end-to-end deep learning methods in classifying a diverse array of diagnoses from raw ECG data (7). Previous research has focused on specific components of the ECG processing workflow, such as localized noise reduction (10) and feature extraction (9), or has been limited to diagnosing specific rhythms, primarily AF and myocardial infarction (MI) (9, 11). This narrow focus contributes to suboptimal accuracy rates in ECG-AI applications and restricts the advancement of AI and deep learning methodologies in this domain (12).

**Figure 1:**
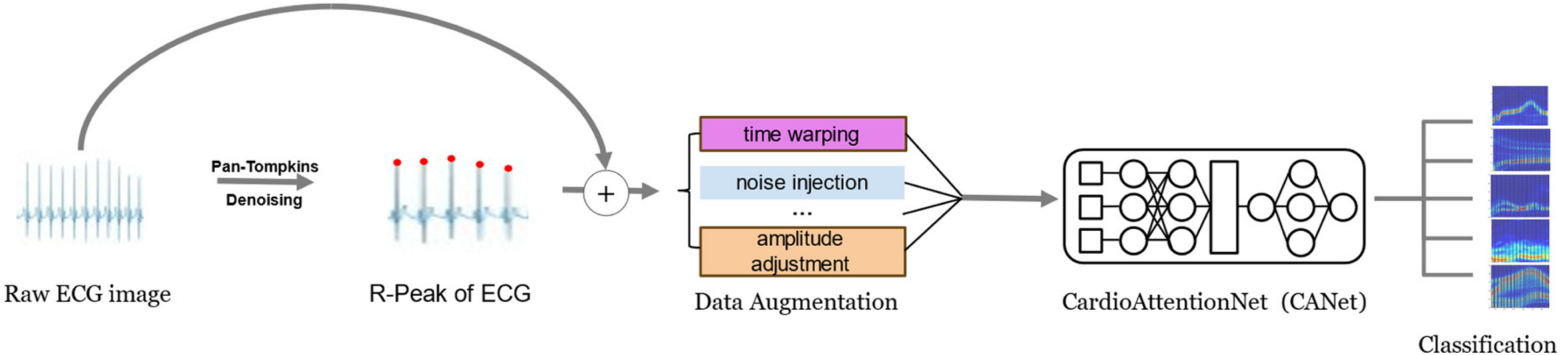
Workflow of this study.

In this study, we address the evolving trends in deep learning and the challenges associated with cardiac arrhythmia detection by introducing the innovative CardioAttentionNet (CANet) model. This model is specifically designed to facilitate portable and accurate arrhythmia detection, thereby enabling early discovery and diagnosis, which can significantly reduce potential harm to patients. CANet is constructed upon the Long Short-Term Memory (LSTM) network and incorporates the Multi-Head Attention mechanism derived from the Transformer model, as well as Depthwise Separable Convolution from MobileNet. A comparative analysis with traditional models indicates that the Bi-directional LSTM (BiLSTM) performs effectively in arrhythmia detection due to its capability to process and retain long-term sequential information, which is essential for analyzing extended rhythm patterns and cyclic variations in electrocardiogram (ECG) signals. However, BiLSTM is limited in its ability to simultaneously focus on multiple features and capture finer details. To overcome this limitation, we have integrated Multi-Head Attention, which allows the model to concentrate on various aspects of ECG signals, in conjunction with Depthwise Separable Convolution for enhanced feature extraction. When compared to baseline models, CANet exhibits superior accuracy and predictive performance, effectively identifying a range of cardiac arrhythmias. The workflow of this study is depicted in Figure 1.

## 2 Methods

### 2.1 Data collection

In this study, we utilized the widely recognized MIT-BIH Arrhythmia Database (13, 14) for our experimental analyses. This database was particularly well-suited for training deep neural networks due to its extensive collection of samples. It consisted of 109,446 electrocardiogram (ECG) recordings, each sampled at a frequency of 360 Hz, which includes both normal heart rhythms and various arrhythmic conditions. Notably, the database segmented these ECG signals into individual beats, each representing a distinct cardiac cycle. Following the standard clinical classification, the heartbeats were categorized into five types: Normal beats (N), Fusion beats (F), Supraventricular ectopic beats (S), Ventricular ectopic beats (V), and Unknown beats (Q). In our research, we adhered to this classification scheme for the identification and analysis of arrhythmias within the ECG signals.

### 2.2 Data preprocessing

Despite the pre-segmentation of electrocardiogram (ECG) signals in the MIT-BIH Arrhythmia Database, these signals continued to exhibit a considerable amount of noise, including electromyographic interference, motion artifacts, and baseline drift, among other factors. Such noise could negatively impact the quality of heart rate signals, thereby hindering the precise extraction of features. Consequently, the preprocessing of these signals was imperative. In the domain of ECG signal preprocessing, wavelet transform was a widely utilized method (15). This technique facilitated the transformation of long-duration time-domain signals into time-frequency representations, thereby elucidating the frequency domain components at each time point and capturing more localized features. During the wavelet transform process, we primarily employed the Mallat algorithm (16). This method systematically decomposed the signal into “low-frequency approximations” and “high-frequency details,” which allowed for the effective removal of frequencies associated with electromyographic noise (20–5,000 Hz) and baseline drift (1 Hz). Following the noise reduction and signal restoration, we achieved ECG signals with improved accuracy and clarity, thereby establishing a robust foundation for subsequent data augmentation processes (17).

**Figure 2:**
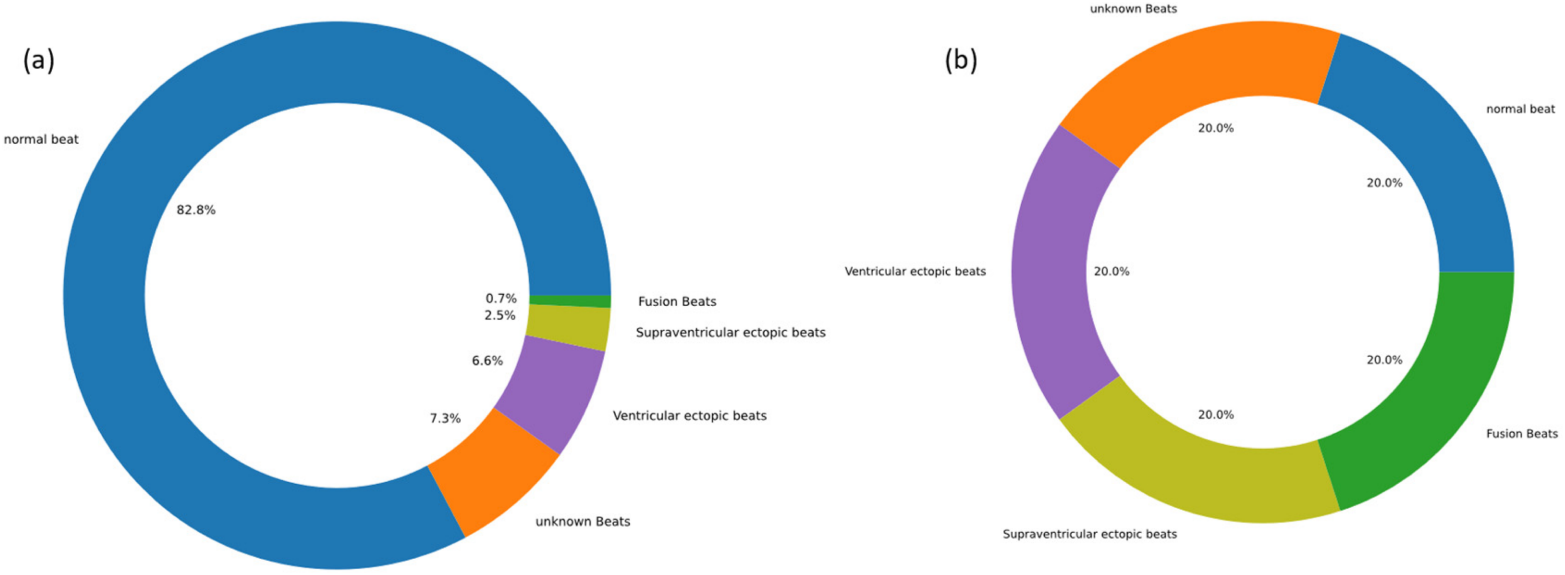
Data display before and after data augmentation. **(a)** before data augmentation. **(b)** after data augmentation.

Recognizing the disparity in the MIT-BIH Arrhythmia Database, with a significant predominance of normal heart rate samples over those exhibiting arrhythmias, we employed data augmentation methods in the preprocessing stage. This approach ensured a balanced representation of all classes in the dataset and bolstered the model's ability to detect abnormal patterns, thus enhancing its overall generalizability. Specifically, we employed data augmentation methods such as time warping and noise injection to create a balanced dataset. These techniques markedly improved the performance of our model, effectively addressing the challenges associated with data imbalance. The adjusted proportions of the different arrhythmia categories in the dataset, both prior to and following data augmentation, were depicted in Figure 2. Experimental results demonstrated that these data augmentation strategies significantly bolstered the model's performance and successfully mitigated the issues related to data imbalance.

### 2.3 Feature extraction

In this study, we selected segmented electrocardiogram (ECG) signals as the primary input, supplemented by each heartbeat interval (R-R interval, RRI) as a parallel input, to establish comprehensive indicators that reflect the key characteristics of cardiac arrhythmias. To enhance accuracy in subsequent deep learning processes, we employed Gaussian filtering to improve data contrast. Although Gaussian filters are predominantly utilized in digital image processing, they are equally effective in enhancing the signal-to-noise ratio, filtering out noise, and ensuring smoother ECG signals (18). Given the waveform deformations frequently observed in ECGs during arrhythmias, we implemented R-wave detection to delineate each individual heartbeat. In our methodology, the Pan-Tompkins algorithm (19, 20) was initially employed to detect R-waves in the denoised ECG signals on a per-minute basis. Subsequently, we calculated the intervals between adjacent R-waves, yielding RRI in milliseconds as a parallel input to the ECG signals, thereby providing a more intuitive representation of the characteristics of cardiac arrhythmias.

### 2.4 Model construction

#### 2.4.1 Cardioattentionnet (CANet)

The advancements in deep learning have significantly enhanced its application in the detection of cardiac arrhythmias, particularly through the utilization of models such as Long Short-Term Memory (LSTM) networks and Recurrent Neural Networks (RNN). These models, especially LSTM, have demonstrated superior recognition accuracy compared to traditional methods (21). LSTM, which represented an improvement over RNN, effectively addresses challenges associated with gradient vanishing and long-term dependencies, thereby enhancing the accuracy of deep learning applications. The efficacy of RNN and LSTM in analyzing time-dependent data was particularly noteworthy, as evidenced by the work of Salloum, Ronald, and Kuo, C.-C. Jay, who reported elevated arrhythmia detection rates using RNN without the necessity for prior feature extraction (22). Furthermore, Hannun et al. (7) introduced a Deep Neural Network (DNN) for classification purposes, underscoring the high sensitivity and predictive capabilities of DNNs. Nonetheless, concerns regarding the accuracy of outputs generated without preprocessing input data persist, highlighting the necessity for a careful balance between accuracy and computational efficiency (21). RNN models retain previous ECG information, influencing current inputs. LSTM units loop information across time steps, creating internal feedback that helps the network understand time and learn temporal dynamics in the data. With these properties, Currently, many models have been using RNN and LSTM models to achieve demonstrated accuracy in arrhythmia detection, including Sumanta et al.'s ELM-RNN model (ECG signal classification and arrhythmia detection using ELM-RNN) and Shu Liu Oh's CNN-LSTM model (Automated diagnosis of arrhythmia using combination of CNN and LSTM techniques with variable length heart beats).

**Figure 3:**
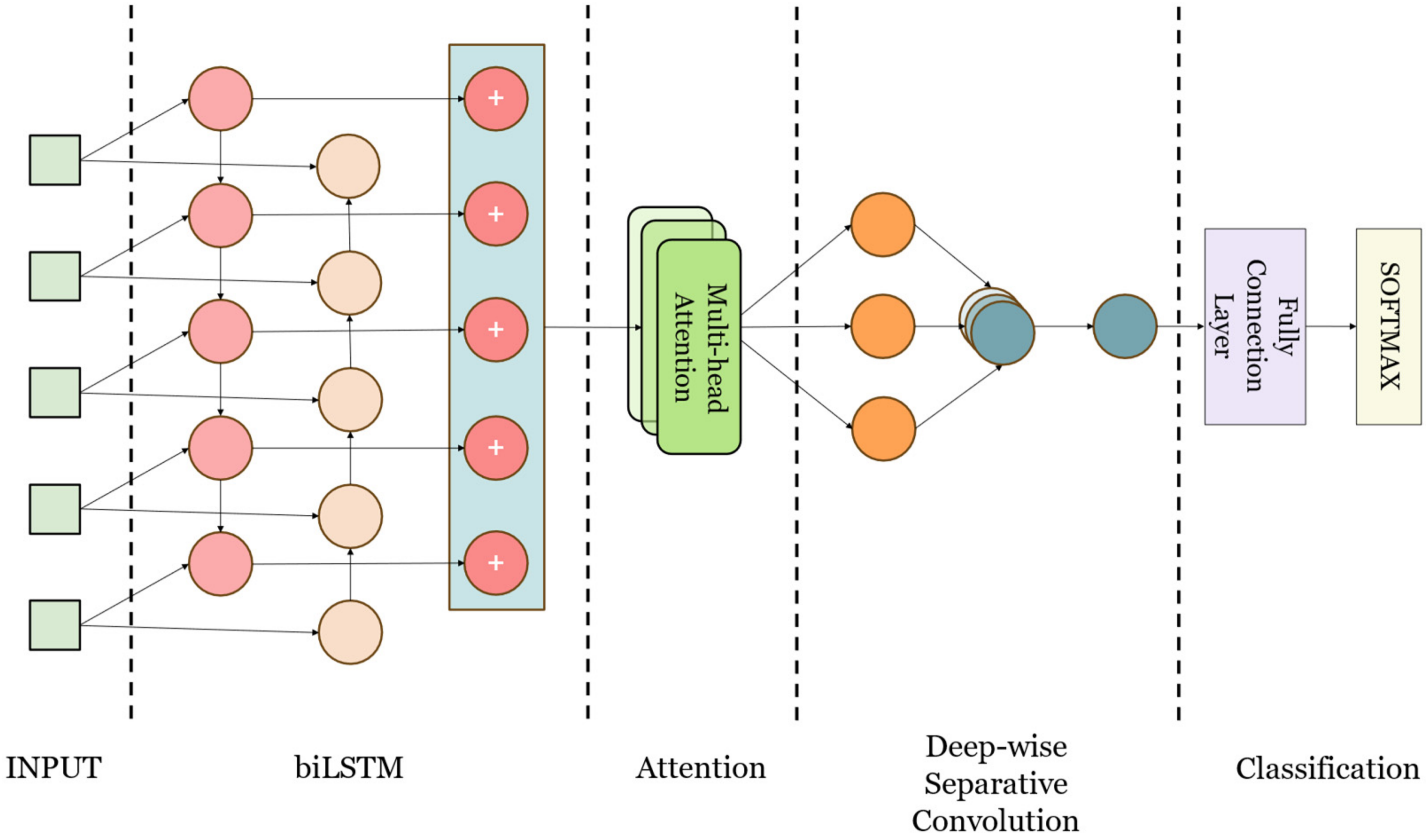
The construction of CANet.

This study presents CANet, a hybrid deep learning model developed for the detection of cardiac arrhythmias. CANet integrates Bidirectional Long Short-Term Memory (BiLSTM), Multihead Attention, and Depthwise Separable Convolution to enhance generalization, predictive performance, and structural robustness, while simultaneously minimizing bias and variance. The design of the model leverages the strengths of various machine learning techniques, amalgamating them into a cohesive framework, as illustrated in Figure 3.

CANet architecture initiates with Bidirectional Long Short-Term Memory (BiLSTM) processing, which enhances temporal dependencies by integrating both past and future data, thereby rendering it appropriate for electrocardiogram (ECG) signal analysis. This configuration facilitates the retention of long-term dependencies, consequently improving classification accuracy. Subsequently, the Multi-Head Attention layer, derived from Transformer models and adapted for image and time series applications, allocates attention to various features concurrently, thereby mitigating the issue of feature neglect (23). This aspect is particularly significant in ECG signal analysis, as it addresses the challenges associated with feature focus in BiLSTM.

The model utilizes Depthwise Separable Convolution, which demonstrates greater efficiency compared to standard convolutions in terms of both parameter count and computational workload, thereby improving training speed (24). This architecture is particularly effective in extracting local time-domain features from electrocardiogram (ECG) signals, which is essential for the identification of abnormal ECG characteristics.

Following comprehensive testing and validation, CANet demonstrates considerable advantages in the detection of cardiac arrhythmias, outperforming conventional models. The architecture incorporates Bidirectional Long Short-Term Memory (BiLSTM) networks, attention mechanisms, and convolutional networks, thereby effectively capturing anomalies in electrocardiogram (ECG) signals from various perspectives. CardioAttentionNet is specifically designed to perform efficiently in data-scarce environments, where smaller datasets are often a reality. Through its lightweight architecture and attention-based feature selection, the model is capable of leveraging limited data effectively, as demonstrated by its strong performance on the MIT-BIH Arrhythmia Database. External evaluations and five-fold cross-validation further substantiate CANet's exceptional performance in arrhythmia detection.

#### 2.4.2 Baseline models

To conduct an objective evaluation of the proposed CANet model, this study established several baseline models for comparative analysis to determine the efficacy and advantages of CANet. The selected baseline models for comparison included Recurrent Neural Networks (RNN), Convolutional Neural Networks (CNN), Long Short-Term Memory networks (LSTM), Bidirectional LSTM (BiLSTM), and Gated Recurrent Units (GRU). These models were chosen due to their prevalent application in deep learning and their significant contributions to research in heart rate detection.

Convolutional Neural Networks (CNNs), a prevalent architecture in deep learning, were recognized for their efficiency in extracting signal features via convolutional operations. This model has gained prominence in the domains of signal classification and segmentation, particularly in the detection of electrocardiogram (ECG) signals, where it has demonstrated a significant impact (25, 26).

Recurrent Neural Networks (RNNs) were acknowledged for their strong model fitting and predictive capabilities when dealing with sequential data, and they have demonstrated significant effectiveness in the detection of arrhythmias. In contrast to conventional deep learning architectures, the hidden layers of RNNs incorporated memory functions that facilitated the retention and utilization of previous information. This characteristic was particularly vital in the analysis of electrocardiogram (ECG) signals (27, 28).

Long Short-Term Memory (LSTM), an extension of Recurrent Neural Networks (RNN), integrates “gates” at each unit (29), thereby enhancing the model's memory capabilities and providing significant advantages in the processing of longer sequences. LSTM has become a widely utilized model in the detection of arrhythmias (30).

The Gated Recurrent Unit (GRU), a variant of the Recurrent Neural Network (RNN) developed to mitigate challenges associated with long-term memory and gradient descent, features a reduced number of “gates” in comparison to the Long Short-Term Memory (LSTM) architecture, thereby improving computational efficiency (31). Furthermore, the GRU has demonstrated significant efficacy in the analysis of electrocardiogram (ECG) signals and related applications.

Bidirectional Long Short-Term Memory (BiLSTM), an enhancement of Recurrent Neural Networks (RNN) and Long Short-Term Memory (LSTM) networks, incorporates a mechanism for the propagation of data in both forward and backward directions. This capability allows BiLSTM to integrate information from both temporal contexts, thereby improving its predictive performance compared to traditional RNN and LSTM architectures (32). Consequently, BiLSTM has attracted significant research interest within the domain of electrocardiogram (ECG) analysis (33–35).

### 2.5 Model evaluation

In order to conduct a thorough and objective assessment of the performance of the proposed CANet model, this study utilized standard evaluation metrics, including Accuracy (ACC), Precision (PRE), Recall (REC), F1-Score (F1), and the Receiver Operating Characteristic (ROC) curve. These metrics were derived from the values of True Positives (TP), True Negatives (TN), False Positives (FP), and False Negatives (FN). The specific formulas for these metrics were outlined as follows:

(1) $$\mathrm{ACC}=\frac{\mathrm{TP}+\mathrm{TN}}{\mathrm{TP}+\mathrm{FP}+\mathrm{TN}+\mathrm{FN}}$$

(2) $$\;\mathrm{PRE}=\frac{\mathrm{TP}}{\mathrm{TP}+\mathrm{FP}}$$

(3) $$\;\mathrm{REC}=\frac{\mathrm{TP}}{\mathrm{TP}+\mathrm{FN}}$$

(4) $$\;F1=\frac{2\times \mathrm{PRE}\times \mathrm{REC}}{\mathrm{PRE}+\mathrm{REC}}$$

where:

• ACC represents the ratio of correctly classified samples to the total number of samples, providing a direct measure of model performance.
• PRE reflects the proportion of actual positive samples that are predicted as positive by the model, indicating the precision of the model. High precision is crucial in applications such as healthcare, where it denotes a lower rate of misclassification.
• REC indicates the proportion of actual positive samples correctly identified by the model, representing the model's coverage. A high recall signifies that the model captures most of the real positive cases.
• The F1 score, the harmonic mean of precision and recall, is an integrated metric, especially important when there is an imbalance between positive and negative samples.

Additionally, the ROC curve graphically represents the performance of a binary classifier, showing the relationship between the True Positive Rate (TPR) and False Positive Rate (FPR) at various threshold settings. Ideally, the best classifier's ROC curve approaches the upper left corner of the graph. The Area Under the Curve (AUC) measures the model's overall ability to distinguish between positive and negative samples. An AUC value close to 1 indicates superior model performance.

## 3 Results

### 3.1 Experiment setup

In this study, comprehensive parameter tuning and optimization were conducted on all developed models to ensure the objectivity and accuracy of the results. To maintain consistency, identical parameter settings were employed throughout the five-fold cross-validation and external testing processes. Iterative experimentation demonstrated that the models converged effectively without overfitting when the number of epochs was set to 30. Specifically, for our CANet model, the learning rate and batch size were fixed at 0.0001, while parameter adjustments were made based on gradient thresholding to enhance model performance.

All experiments were conducted on a platform running Windows 11 Professional operating system, with Python 3.10.9 as the runtime environment. Regarding software libraries, Pytorch 2.0.1 + cu117, Scikit-learn, Sklearn 0.0.post1, scipy 1.10.0, and other mathematical libraries were utilized to support the construction of the model structure and the validation of results. The hardware configuration included an Intel Core i7 10750H processor (base frequency of 2.6 GHz, turbo frequency up to 5 GHz, 6 cores/12 threads) and an NVIDIA GeForce GTX 1080Ti graphics card (8 GB memory capacity, 128-bit memory bus width).

### 3.2 Result of five-fold cross-validation

**Figure 4:**
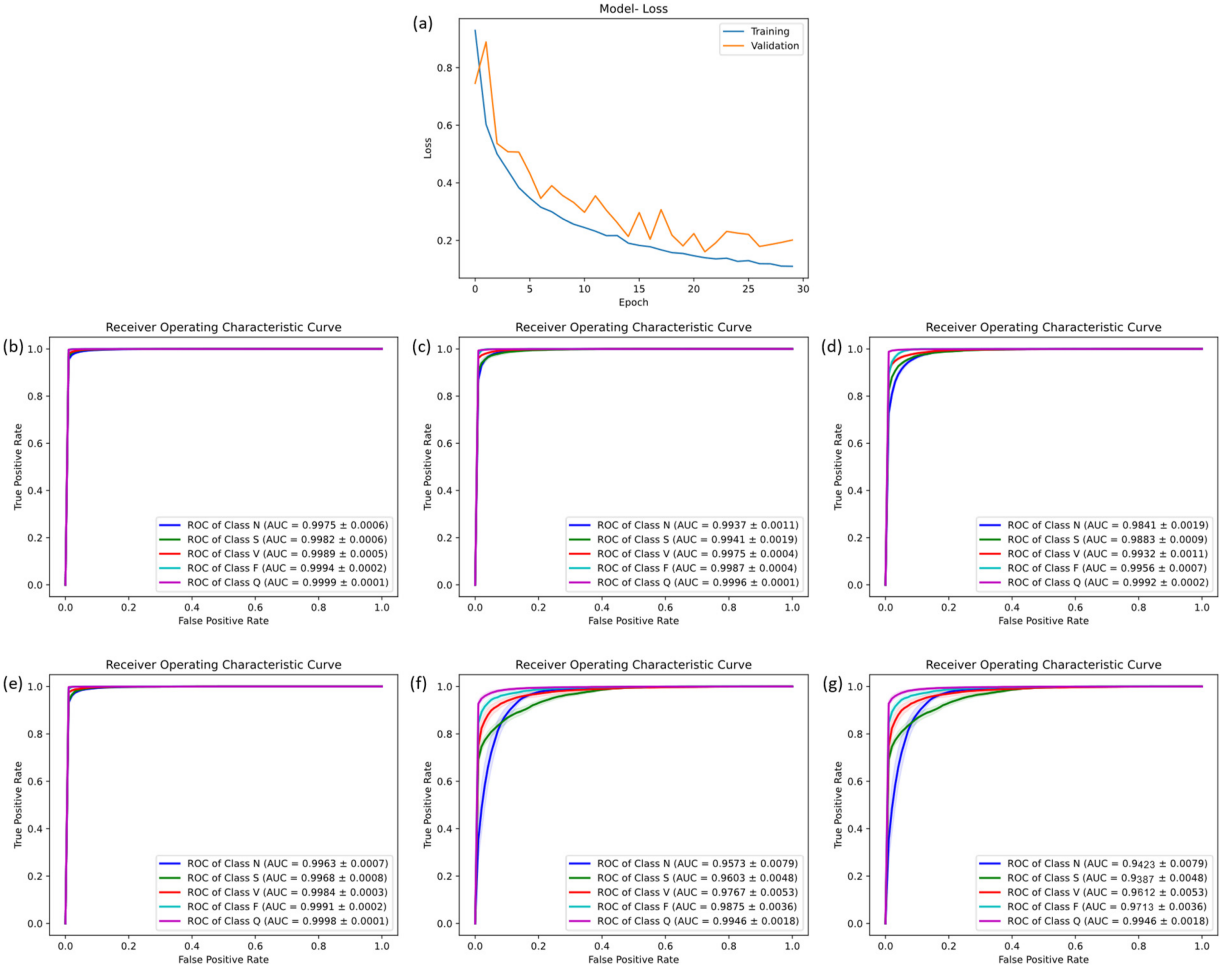
The result of five-fold cross-validation. **(a)** the training flow of CANet. **(b)** The ROC curve of CANet. **(c–g)** The ROC curve of BiLSTM, CNN, GRU, LSTM and RNN.

To comprehensively and objectively evaluate the performance of the proposed CANet model, this study utilized the five-fold cross-validation method for dataset division and conducted rigorous testing of the model. The mean values of the model test results were used to reduce randomness and improve generalization capability. After 10 training epochs, optimal performance for CANet was observed with a learning rate of 0.0001, which helps to ensure that the model's weights are updated in small steps, preventing large oscillations or divergence in the loss function. To provides a good balance between accurate gradient estimation and computational efficiency, the batch size is 64, without overfitting. The final training iteration graph is depicted in Figure 4a.

During the five-fold cross-validation, CANet showed a performance higher than all other models. In the accuracy scale, this model shown a stable ability of prediction, which was the only one that recognized all five heartbeats with a accuracy higher than 95%. Moreover, it outperformed all baseline models across all evaluation matrics (ACC, PRE, REC and F1), showcasing exceptional predictive performance.

These results highlight CANet's high accuracy in recognizing five different types of heartbeats. The corresponding ROC curves and confusion matrices are shown in Figures 4b,c.

Compared to the five baseline models, CANet showed more favorable results in the cross-validation. The baseline models' ACC, PRE, REC, and F1 scores were lower than CANet's, which is shown in the Supplementary Table S1.

Among them, the GRU model performed the best, which mean ACC reached 97%, but its precision was significantly lower than CANet. The BiLSTM model slightly trailed GRU in classification accuracy. The performance of the remaining models declined further across several metrics. Overall, traditional models fell short in various metrics compared to CANet, which demonstrated a clear advantage in the cross-validation. The ROC curves for the baseline models are presented in Figures 4c–g.

### 3.3 Result of external validation

**Figure 5:**
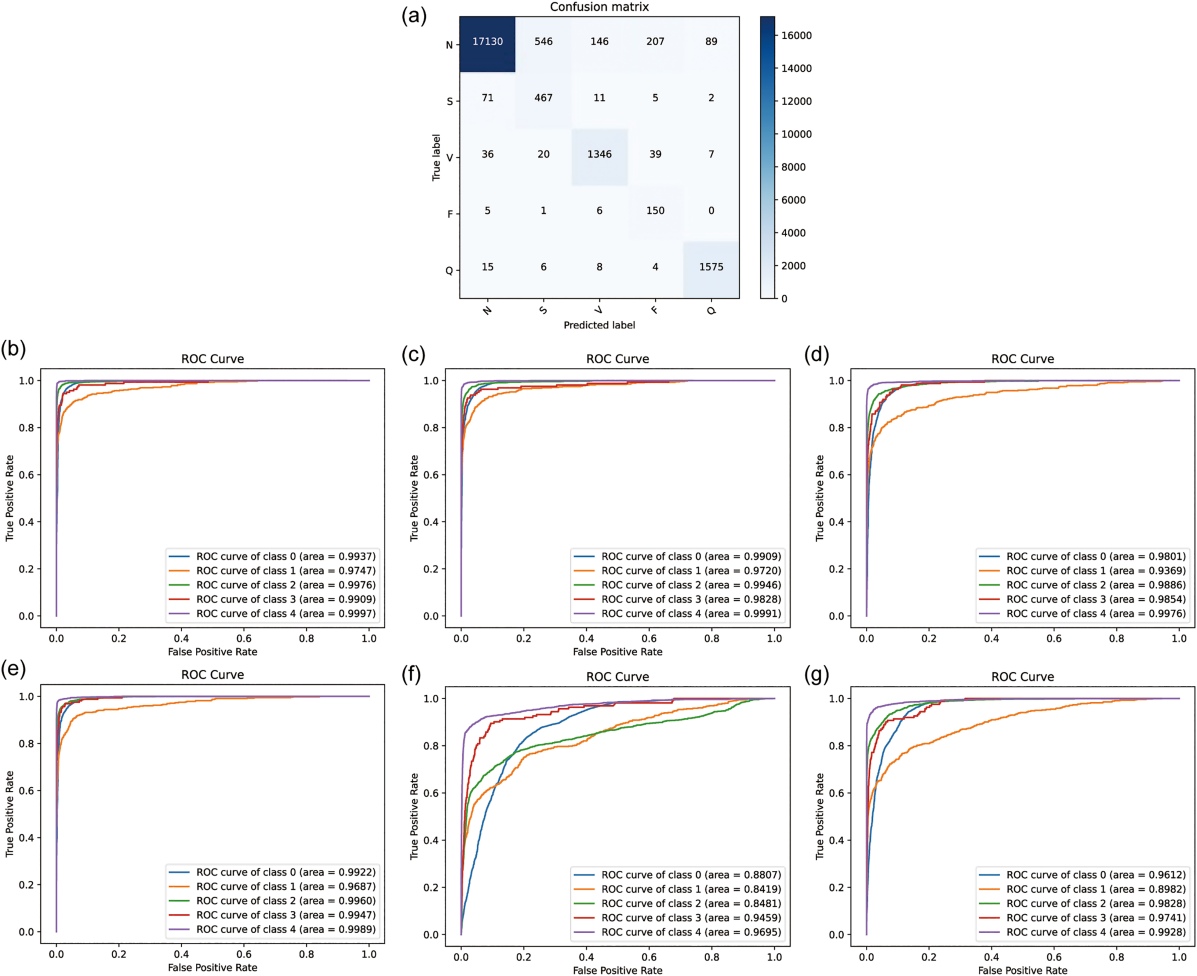
The result of external validation. **(a)** the confusion matrix of CANet. **(b)** The ROC curve of CANet. **(c–g)** The ROC curve of BiLSTM, CNN, GRU, LSTM and RNN.

To evaluate the performance of the newly developed CANet model, an external test using an unseen sample set was conducted to assess its capability in handling unseen data. The results demonstrate that CANet performed exceptionally well, with average ACC, PRE, REC, and F1 of 94.41%, 92.41%, 92.41%, and 79.02%, respectively. Notably, the model accurately identified [S] and [Q] type, achieving precision rates of 99.26% and 99.22%, respectively. For [N] type, CANet consistently showed reliable performance across all metrics, with ACC, PRE, REC, and F1 at 94.91%, 99.26%, 94.55%, and 96.85%, respectively. Additionally, the model was trained and tested using data prior to data augmentation, yielding average ACC and PRE of 93.27% and 87.23%, respectively. Due to imbalances in the dataset, a significant number of [Q] type predictions were incorrectly classified as [N], markedly reducing precision. These findings underscore the significant improvement in model performance facilitated by data augmentation. Overall, CANet maintained high accuracy and precision across different types of heartbeats without significant performance variation. The corresponding confusion matrix and ROC curve are presented in Figures 5a,b.

In external testing of baseline models, the GRU model exhibited the highest accuracy, with mean ACC, PRE, REC, and F1 of 93.76%, 73.06%, 93.14%, and 79.39%, respectively. Despite its high recognition rate, GRU's performance across all evaluation metrics was inferior to CANet, especially in PRE and F1. The performance of all models in the external test is detailed in Supplementary Table S2.

Other baseline models demonstrated lower precision, accuracy, recall, and F1-scores. Additionally, GRU showed lower precision in recognizing [S] and [V] types, with rates of only 38.38% and 41.86%, respectively. The accuracy of BiLSTM was slightly lower than GRU, at 91.74%, with a precision of only 67.43%. The remaining models (LSTM, CNN, RNN) showed poorer recognition results, with lower accuracy, precision, recall, and F1. The results indicate that baseline models were inferior to CANet in terms of accuracy and adaptability to the dataset, displaying a clear imbalance in recognizing different types of heartbeats. Additionally, the ROC curves of the baseline models are illustrated in Figures 5b–g. Analysis of these curves reveals that the CANe model surpasses all baseline models in AUC values for each classification. Apart from the [S] category, which has an AUC of 97.47%, all other classifications have AUCs exceeding 99%, demonstrating exceptional clinical predictive value. These results confirm the superior predictive performance of the CANe model. Given its lightweight design, the model is poised for widespread deployment in clinical electrocardiographic devices and portable wearable devices, facilitating precise and real-time detection of cardiac arrhythmias.

We measured the inference time for the model to process a single ECG signal on a standard hardware platform (Intel Core i7 CPU with 16 GB RAM). The average inference time was found to be approximately 56.7 ms, which is well within the typical requirement for real-time detection. This result is consistent with the claim that the model can be used for real-time arrhythmia detection. The model was found to require approximately 30 MB of memory during inference. This low memory footprint demonstrates the portability of CardioAttentionNet, making it feasible for deployment on resource-constrained devices, such as wearable ECG monitors or mobile health applications.

## 4 Discussion

In this study, we proposed an innovative fusion model referred to as CANet, which integrates Bidirectional Long Short-Term Memory (BiLSTM), Multihead Attention, and Depthwise Separable Convolution. This model was specifically designed to achieve high accuracy and robustness while maintaining a lightweight architecture. CANet is employed for the classification of electrocardiogram (ECG) signals, with the objective of distinguishing abnormal heartbeats from normal cardiac rhythms, thereby facilitating the detection of cardiac arrhythmias. The results indicated that CANet exhibits remarkable recognition capabilities for five clinically distinct types of heartbeats. During five-fold cross-validation, CANet achieved high average precision, with 95% confidence intervals for the various heartbeat types as follows: Normal (N) 97.92 ± 0.33%, Supraventricular (S) 98.58 ± 0.21%, Ventricular (V) 99.13 ± 0.13%, Fusion (F) 99.30 ± 0.17%, and QRS (Q) 99.74 ± 0.5%. In external testing, CANet continued to demonstrate excellent performance, attaining average accuracy (ACC), precision (PRE), recall (REC), and F1 scores of 94.91%, 99.26%, 94.55%, and 96.85%, respectively.

Although the CANet model demonstrated superiority across multiple tasks in this study, we also acknowledge that other models retain advantages in specific scenarios. The GRU model, in particular, excels in handling time-series data, especially in situations characterized by strong short-term dependencies. Due to its streamlined architecture and lower computational complexity, the GRU may offer higher efficiency in environments with limited resources or computing power. In contrast, while CANet boasts more robust performance, its complexity may render it less suitable for scenarios sensitive to computational speed or resource demands. Moreover, despite CANet's demonstrated higher accuracy and robustness in our investigated tasks, lightweight models such as the GRU might still be viable choices in tasks or with data features where these characteristics are prominent. These models could have advantages in applications requiring real-time responses or operating under resource constraints. Therefore, the selection of models in practical applications should consider the specific task requirements, computational resources, and data characteristics comprehensively.

The exemplary performance of CANet can be attributed to the synergistic combination of its architectural design and predictive capabilities. The integration of LSTM, Multi-Head Attention, and Depthwise Separative Convolution enables effective processing and memory of long-term sequential data, identification of cyclic patterns in ECG signals, and simultaneous focus on multiple features. Additionally, the temporal nature of cardiac signals offers an ideal setting for the BiLSTM component of the model, yielding more precise and efficient recognition capabilities. Through this optimized design, we have significantly enhanced the model's performance while minimizing its parameters, thus avoiding excessive computational load on the hosting devices. As a result, the model holds potential for future deployment in portable devices for real-time and precise detection and classification of cardiac arrhythmias. These combined elements contribute to the outstanding performance of CANet, showing potential for application in wearable devices and clinical practice for early arrhythmia diagnosis, thereby mitigating its impact on health.

**Figure 6:**
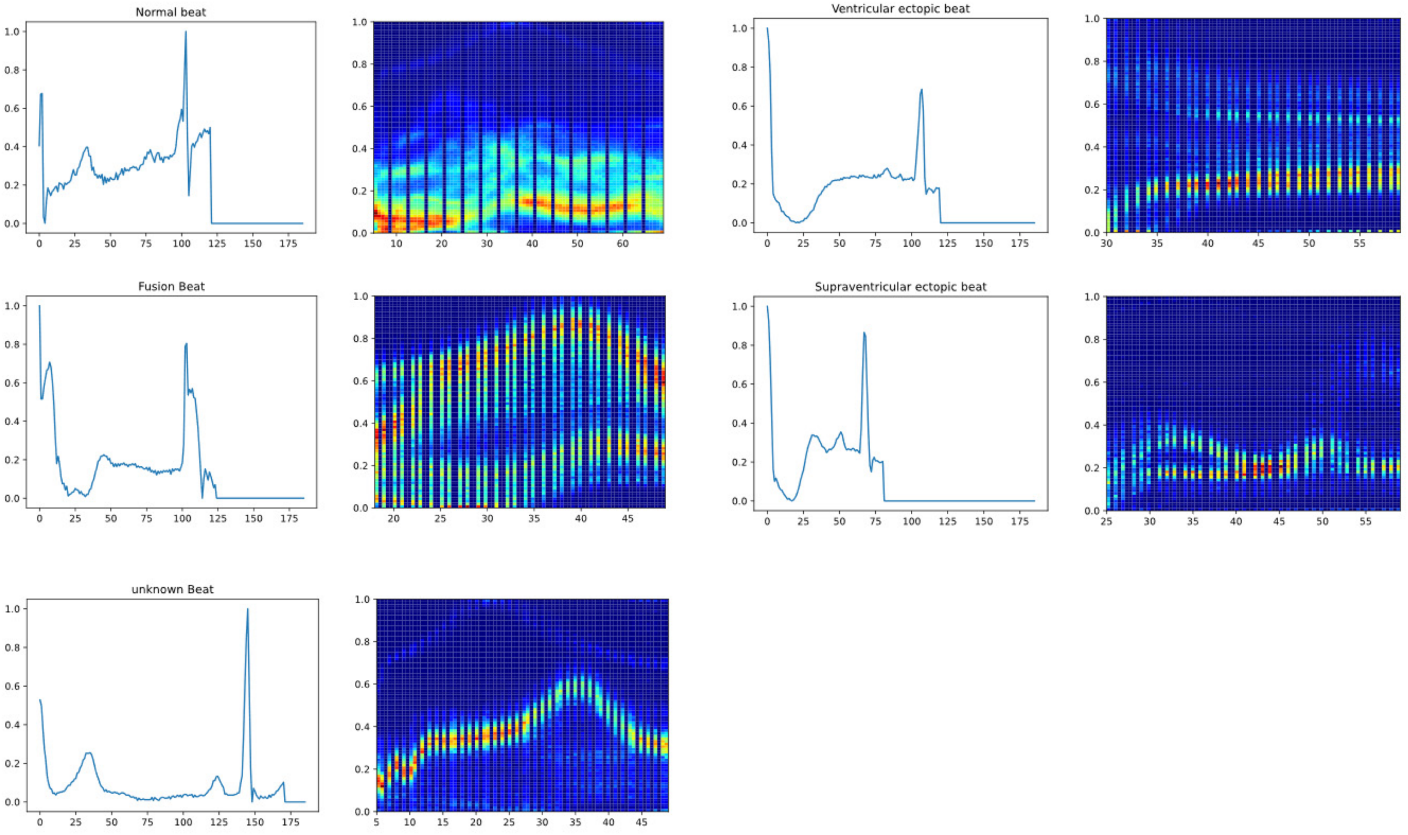
The heat activation map of new model (From left to right, up and down are respectively normal beat, ventricular ectopic beat, fusion beat, supraventricular ectopic beat, unknown beat).

To illustrate the model's feature extraction and effective recognition of ECG signals, a heat activation map is presented as a visualization of the model output in Figure 6. Specifically, for Normal beats (N), the heatmap shows significant activation during the QRS complex, indicating accurate identification of this critical interval with reduced activation during the T wave, consistent with normal electrophysiological characteristics. For Fusion beats (F), the activation pattern is uniformly distributed during the QRS complex and *P* wave, reflecting variations in cardiac signals in these regions, corresponding to the clinically observed shortened interval between the *P* wave and QRS complex. For Supraventricular ectopic beats (S), the heatmap exhibits heightened activity at the onset of the QRS complex, suggesting the model's sensitivity to changes in QRS morphology typical of supraventricular ectopy, aligning with the clinical presentation of abnormal *P* waves and altered QRS morphology. For Ventricular ectopic beats (V), the clinical presentation of wide and abnormal QRS complexes without preceding *P* waves is mirrored by intense activity throughout the QRS interval on the heatmap, consistent with the characteristics of ventricular ectopy. For Unknown beats (Q), significant activation is shown on the *T* wave following the QRS complex, indicating that the model can effectively capture features of abnormal waveforms when dealing with uncertain categories. This visual representation demonstrates CANet's ability to differentiate between various heart rate variabilities, emphasizing the model's interpretability and potential to enhance trust among medical professionals and patients.

The electrocardiogram (ECG) serves as an essential tool in the routine practice of clinical medicine, with over 300 million ECGs conducted worldwide each year (36). Arrhythmias are highly prevalent; however, the challenges associated with monitoring certain malignant arrhythmias contribute to elevated mortality rates. The enhanced accuracy in diagnosing cardiac arrhythmias is primarily attributed to the expertise of clinicians, a situation that is increasingly untenable given the current global shortage of specirealized cardiologists. For instance, China has only 4.8 cardiologists per 100,000 individuals (37), while Japan is reported to have approximately 14,000 specialized cardiologists (38), figures that are grossly insufficient relative to their large populations. In response to these challenges, wearable devices incorporating deep learning technologies are being increasingly integrated into daily life (39). Research indicates that the Apple Watch, the most widely utilized wearable device, exhibits a sensitivity of only 25% for atrial flutter/atrial tachycardia (AFL/AT) (40), while the Fitbit demonstrates even lower accuracy in diagnosing atrial arrhythmias (41). The purpose of this study is to develop an ECG-AI model that accurately detects arrhythmia. Compared to traditional methods, this deep learning approach eliminates the need for manual design and selection of features, significantly reducing the expertise and time required for arrhythmia diagnosis. Moreover, by diminishing the reliance on specialized knowledge, the model can be deployed on everyday wearable devices such as the Apple Watch to facilitate routine monitoring of arrhythmias. This will assist clinicians in obtaining more precise ECG diagnoses and providing technical support for wearable and hospital devices.

In recent years, many studies have explored the application of deep learning models in arrhythmia classification, some of which focused on the problem of five categories of arrhythmias. Although these studies have demonstrated the potential of AI in arrhythmia detection, existing models vary significantly in complexity, accuracy, and computational efficiency. Our proposed CANet model significantly improves on traditional methods, especially in terms of lightweight design, real-time detection capabilities, and portability, which are critical for the clinical application of wearable devices. For example, Attia et al. (8) proposed an AI-assisted ECG algorithm for identifying atrial fibrillation (AF) in sinus rhythm with an accuracy of 83.3%. However, the study only focused on one type of arrhythmia, while our model solves the problem of multi-class classification and can distinguish between normal, atrial, ventricular, atrial fibrillation, and unclassified arrhythmias, providing a more comprehensive solution. In addition, CANet integrates a multi-head attention mechanism that can simultaneously focus on multiple features of the ECG signal, improving accuracy without increasing computational complexity, which is also a limitation of many existing models. Similarly, Faour et al. (11) studied a convolutional neural network (CNN)-based AI model for detecting ST-segment elevation myocardial infarction. Although the model was effective in detecting specific types of arrhythmias, its high model complexity limited its application on portable devices. In contrast, CANet significantly reduced the number of parameters and improved computational efficiency by using deep separable convolutions, making it suitable for real-time arrhythmia detection on wearable ECG devices. Finally, the deep neural network (DNN) developed by Hannun et al. (7) showed high sensitivity and specificity in classifying arrhythmias from raw ECG data. Although this method performed strongly, its application in portable devices was limited by its model complexity and large size. Our model maintains high performance while adopting a lightweight design, enabling real-time detection and making it more suitable for deployment in resource-constrained environments. Compared with these studies, CANet not only achieves competitive accuracy, but also further improves performance without increasing computational overhead by integrating an efficient architecture and attention mechanism to optimize feature extraction. This makes it particularly suitable for clinical environments that require real-time continuous monitoring and portability. In addition, CANet uses data augmentation techniques such as time warping and noise injection to enable it to perform well even with limited data, which is a scenario that many existing models have difficulty handling.

Of course, there are some limitations to this study. Although the model demonstrated superior accuracy compared to others during validation, it still presents a risk of misdiagnosis, particularly in the classification of ventricular ectopic beats and normal beats, where accuracy is lower. This suggests that the model's effectiveness in arrhythmia detection may require further enhancement through strategies such as data augmentation and balancing the dataset by oversampling rare classes or undersampling common ones to reduce bias towards specific patterns. As this study focuses on the development of deep learning methodologies, and given the limitations of dataset size and volume, comprehensive testing across a broad spectrum of cardiac arrhythmia classifications was not conducted. Consequently, the training and validation of this study have certain limitations. Given the current model's superior classification performance, we plan to incorporate additional data in future work and train the model to recognize a wider array of arrhythmia types, thus broadening its applicability. Additionally, the model's external testing process has certain limitations: while external validation typically requires testing across data from diverse sources, this model's external test was performed on a held-out set from the MIT-BIH dataset, which introduces some bias and reduces the model's robustness. Furthermore, this study was not tested across extensive datasets, diverse populations, or complex clinical settings. Therefore, it is necessary to further evaluate the model's performance using datasets with broader coverage. Specifically, the current publicly available database used can only detect the types of arrhythmias mentioned earlier, and in the future, we plan to expand to include additional relevant types. Additionally, there is a lack of external test data in this study, and we may conduct a multi-center study to improve this in the future. Lastly, the system has not been developed or deployed yet, and further development is required for clinical translation.

## 5 Conclusions

In this endeavor, researchers have created an AI-powered ECG model. The model has superior performance in both five-fold cross-validation and external validation compared to other models, indicating its greater feasibility and potential for reliably detecting arrhythmias through a fusion deep learning model featuring separable convolution. The principle aim of the study is to facilitate the procurement of more precise ECG diagnoses through technological advancement and to augment technical support for wearable and clinical devices utilized in clinical circumstances.

## Data Availability

The datasets presented in this study can be found in online repositories. The names of the repository/repositories and accession number(s) can be found in the article/Supplementary Material.

## Supplementary material

Supplementary Table S1 Result of five-fold cross validation.

Supplementary Table S2 Result of external validation.
